# The Association between a Decrease in On-Treatment Neutrophil-to-Eosinophil Ratio (NER) at Week 6 after Ipilimumab Plus Nivolumab Initiation and Improved Clinical Outcomes in Metastatic Renal Cell Carcinoma

**DOI:** 10.3390/cancers14153830

**Published:** 2022-08-07

**Authors:** Yu-Wei Chen, Matthew D. Tucker, Landon C. Brown, Hesham A. Yasin, Kristin K. Ancell, Andrew J. Armstrong, Kathryn E. Beckermann, Nancy B. Davis, Michael R. Harrison, Elizabeth G. Kaiser, Renee K. McAlister, Kerry R. Schaffer, Deborah E. Wallace, Daniel J. George, W. Kimryn Rathmell, Brian I. Rini, Tian Zhang

**Affiliations:** 1 Division of Hematology Oncology, Vanderbilt University Medical Center, 1211 Medical Center Drive, Nashville, TN 37232, USA; 2 Grandview Cancer Center, Alabama Oncology, 3670 Grandview Pkwy, Birmingham, AL 35243, USA; 3 Levine Cancer Institute, Atrium Health, 1021 Morehead Medical Drive, Charlotte, NC 28204, USA; 4 Duke Cancer Institute, 2 Seeley Mudd, 10 Bryan Searle Drive, Durham, NC 27710, USA; 5 Division of Hematology Oncology, UT Southwestern Medical Center, 5323 Harry Hines Blvd., Dallas, TX 75390, USA

**Keywords:** immune checkpoint inhibitor, eosinophil, neutrophil-to-eosinophil ratio, NER, renal cell carcinoma, kidney cancer

## Abstract

**Simple Summary:**

Immune checkpoint inhibitors (ICIs) have significantly changed the treatment paradigm in metastatic renal cell carcinoma (mRCC) and brought an unprecedented durable response. However, there is still a significant proportion of patients who do not response to ICIs, and there are no biomarkers to select responders. In this study, we investigated the change in neutrophil-to-eosinophil ratio (NER) during ipilimumab/nivolumab treatment and clinical response in mRCC. We found that mRCC patients who responded to immunotherapy had lower on-treatment NER during ipilimumab/nivolumab induction. In addition, after accounting for baseline tumor biological characteristics and patient sociodemographic factors, we found that the decrease in NER at week 6 was independently associated with improved outcomes in ipilimumab/nivolumab-treated mRCC. Given that the NER can be easily obtained through routine laboratory work-ups, our results provide initial evidence that the decrease in on-treatment NER during immunotherapy, as a biomarker to predict ICI treatment response, warrants further investigation in prospective studies.

**Abstract:**

A lower baseline neutrophil-to-eosinophil ratio (NER) has been associated with improved responses to immune checkpoint inhibitors (ICI)-treated metastatic renal cell carcinoma (mRCC). This study investigated the decrease in NER at week 6 after ipilimumab/nivolumab (ipi/nivo) initiation and treatment responses in mRCC. A retrospective study of ipi/nivo-treated mRCC at two US academic cancer centers was conducted. A landmark analysis at week 6 was performed to assess the association between the change in NER and clinical responses (progression-free survival (PFS)/overall survival (OS)). Week 6 NER was modeled as a continuous variable, after log transformation (Ln NER), and a categorical variable by percent change. There were 150 mRCC patients included: 78% had clear cell histology, and 78% were IMDC intermediate/poor risk. In multivariable regression analysis, every decrease of 1 unit of Ln NER at week 6 was associated with improved PFS (adjusted hazard ratio (AHR): 0.78, *p*-value:0.005) and OS (AHR: 0.67, *p*-value: 0.002). When NER was modeled by percent change, decreased NER > 50% was associated with improved PFS (AHR: 0.55, *p*-value: 0.03) and OS (AHR: 0.37, *p*-value: 0.02). The decrease in week 6 NER was associated with improved PFS/OS in ipi/nivo-treated mRCC. Prospective studies are warranted to validate NER change as a biomarker to predict ICI responses.

## 1. Introduction

Immune checkpoint inhibitors (ICIs) targeting program-cell death 1 or its ligand (anti-PD1/anti-PDL1) and cytotoxic T-lymphocyte associated protein 4 (anti-CTLA-4) have revolutionized the treatment paradigm for multiple cancers and have brought unprecedented deep and durable treatment efficacy to patients with metastatic disease [1,2,3,4,5]. Kidney cancer is one of the leading examples demonstrating how immune checkpoint inhibitors enable sustained long-term overall survival in metastatic disease: after a minimal 5-year follow-up of the CheckMate 214 trial [6], almost 50% of the ipilimumab/nivolumab treated metastatic clear cell renal cell carcinoma (mccRCC) patients were still alive, with a median overall survival of 56 months, compared to that of 38 months in the sunitinib arm among the intention-to-treatment population. In addition, the median duration of response has not yet been reached.

Despite the great success and ongoing efforts in developing immuno-oncology (IO) agents [7], there is a significant fraction of patients who will not respond to IO, and there is a need to develop predictive biomarkers to improve patient selection. The search for biomarkers has led to enhanced understanding of the tumor microenvironment (TME) in IO-treated patients [8,9]: studies have elucidated the complex interactions between infiltrating immune cells, tumor cells, and other surrounding cell, such as fibroblasts and endothelial cells [9,10]. While infiltrating T lymphocytes caught the early attention of researchers in the studies of TME [11], there has been rapidly accumulating evidence supporting the roles of myeloid cells [12,13] in the response of ICIs, such as the myeloid-derived suppressor cells and tumor-associated macrophages [14]. Furthermore, there is growing evidence among pre-clinical studies suggesting that eosinophils are essential cells in the TME for the anti-tumor activity observed with IO [15,16]. Recent clinical studies also reported that a higher baseline eosinophil count was predictive of improved ICI responses in melanoma [17,18], urothelial cancer [19], and head and neck cancer [20]. Besides the predictive value of the baseline eosinophil count, the interval increase in eosinophil count after initiation of ICIs was also observed among responders in lung cancer [21,22], melanoma [23], and kidney cancer [24].

Our group previously investigated the baseline neutrophil-to-eosinophil ratio (NER) in ipilimumab/nivolumab treated mccRCC and demonstrated that a low baseline NER was associated with favorable progression-free survival (PFS), overall survival (OS), and objective response rate (ORR) [25]. Similar improved outcomes were also observed in nivolumab monotherapy-treated mRCC at second or later lines of therapy [26]. Using NER as a predictive biomarker is clinically pertinent to RCC, since neutrophilia is a well-validated International Metastatic RCC Database Consortium (IMDC) prognostic factor [27,28]. Another advantage of adopting a ratio is to reduce the interlaboratory variability of different assays compared to using absolute eosinophil count alone. Building on our prior findings of the favorable prognostic/predictive value of low baseline NER [25], we investigated the dynamic changes of NER during the induction phase of ipilimumab/nivolumab from baseline to week 12 and the association between the on-treatment change of NER with clinical outcomes in ipilimumab/nivolumab-treated mRCC.

## 2. Methods

### 2.1. Patient Population

Patients diagnosed with mRCC and treated with the combination of ipilimumab/nivolumab at Vanderbilt-Ingram Cancer Center and Duke Cancer Institute were identified. Patients who had received prior immune checkpoint inhibitors before ipilimumab/nivolumab were not eligible for inclusion. The study population included patients treated between January 2016 to 21 August 2021, January 2016 to 26 March 2021, for patients treated at Vanderbilt and Duke, respectively. All investigators had access to the study population. This study received Institutional Review Board approval from each institute.

### 2.2. Variables and Endpoints of Interests

The main variable of interest was the neutrophil-to-eosinophil ratio (NER), which was calculated by the absolute neutrophil count (ANC, cell number × 10^3^/µL) divided by the absolute eosinophil count (AEC, cell number × 10^3^/µL). The NER measurements included baseline and throughout the first 12 weeks of the ipilimumab induction (week 3, 6, 9, and 12).

Patient sociodemographic factors included age, race (White, versus non-White), and sex. Tumor characteristics included histology type (clear cell RCC (ccRCC), versus non-clear cell RCC (nccRCC)), and the IMDC risk group [27] (favorable, intermediate, and poor risk). Prior treatment information included nephrectomy (Yes or No) or systemic therapy (Yes or No).

The endpoints of interest were the length of overall survival (OS), progression-free survival (PFS), and objective response rate (ORR) [29].

### 2.3. Statistical Analysis

Patient sociodemographic factors, tumor characteristics, and prior treatment information were presented with descriptive statistics. Categorical variables were compared using the chi-square test (or Fisher’s exact test, if cell count ≤ 5). The Mantel–Haenszel chi-square test was used to report *p*-value for trend. The Wilcoxon rank sum test was used to compare AEC, ANC, and NER between responders and non-responders.

Landmark analysis was conducted to investigate the association between on-treatment NER at week 6 and PFS/OS. PFS was calculated from week 6 after treatment initiation until clinical/radiographic progression or death. OS was calculated from week 6 after treatment initiation until death. Given the non-normal distribution of NER, natural log transformation of NER (LnNER) was used in continuous variable analysis. For categorical variable analysis, relative NER change at week 6 from baseline was categorized into three groups (increase, ≤50% decrease, >50% decrease). Multivariable Cox regression analysis was used to assess the association between week 6 NER with PFS or OS after baseline risk adjustments. The Kaplan–Meier method was used to present PFS and OS. A two-sided *p*-value < 0.05 was considered statistically significant. All statistical analyses were performed using SAS version 9.4 (SAS Institute Inc., Cary, NC, USA), and survival curves were plotted with GraphPad Prism version 9.0 (GraphPad Software Inc., San Diego, CA, USA).

## 3. Results

### 3.1. Changes in AEC/ANC/NER and Patient Baseline Characteristics

**Figure 1:**
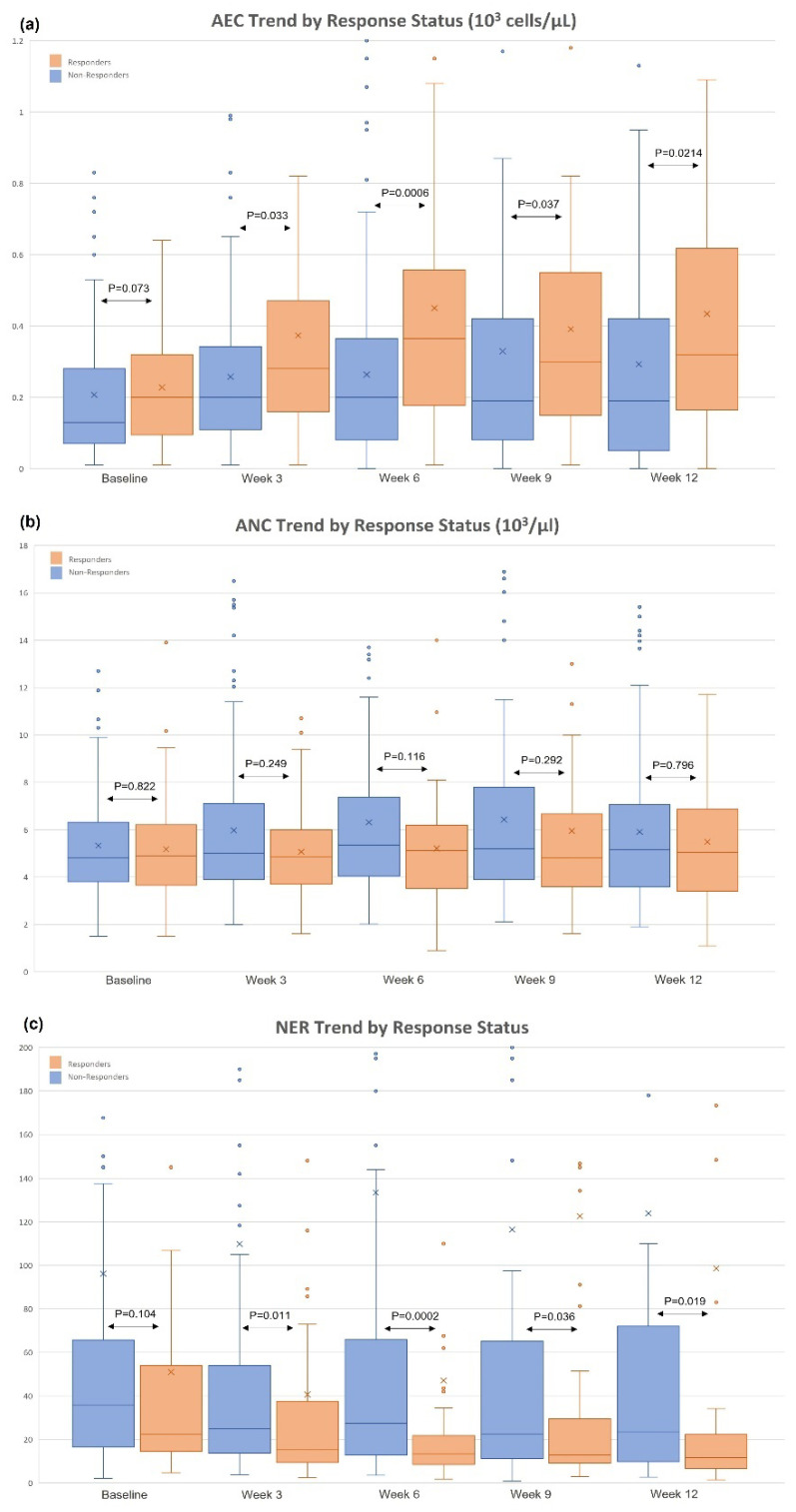
Trend of AEC/ANC/NER by response status.

The initial study population consisted of 166 patients with mRCC treated with ipilimumab/nivolumab. The trend of AEC, ANC, and NER from baseline to week 12 were stratified by response status (Figure 1). Among responders, the median AEC was 200 cells/µL at baseline, 365 cells/µL at week 6, and 320 cells/µL at week 12; among non-responders, the median AEC was 130 cells/µL at baseline, 200 cells/µL at week 6, and 190 cells/µL at week 12. The most significant AEC difference was observed at week 6 (Figure 1a; *p*-value: 0.0006). For ANC, there was no statistical difference at either time point (Figure 1b; *p*-values > 0.05). The median NER was numerically lower in responders from baseline to week 12 (Figure 1c). The most significant difference of NER was observed at week 6 (Figure 1c; *p*-value: 0.002). Given the above observations, the week 6 NER was further investigated in landmark analyses. The final cohort included 150 patients with mRCC treated with ipilimumab/nivolumab (10 patients with missing week 6 NER, and 6 patients progressed before week 6 were excluded).

There were 150 ipilimumab/nivolumab-treated patients with mRCC included in the final study population (Table 1). The median age was 62 (interquartile range: 53–70), 74% were male, and 80% were White. The majority of patients had clear cell histology (78%); 22% were IMDC favorable risk, 63% were intermediate risk, and 15% were poor risk. There were 104 (69%) patients who had prior nephrectomy, and 49 (33%) patients who had prior systemic therapy. The median follow-up time was 11.6 months.

### 3.2. Association between Decreased NER at Week 6 and Clinical Outcomes

In the multivariable Cox regression analysis, NER was first modeled as a continuous variable after natural log transformation. After adjusting for age, sex, race, IMDC risk group, baseline LnNER, histology, prior systemic therapy, and prior nephrectomy, every decrease of 1 unit of week 6 LnNER was associated with improved PFS (adjusted hazard ratio (AHR):0.78, 95% CI: 0.66–0.93, *p*-value: 0.005) and OS (AHR:0.67, 95% CI: 0.52–0.86, *p*-value: 0.002) (Table 2).

**Figure 2:**
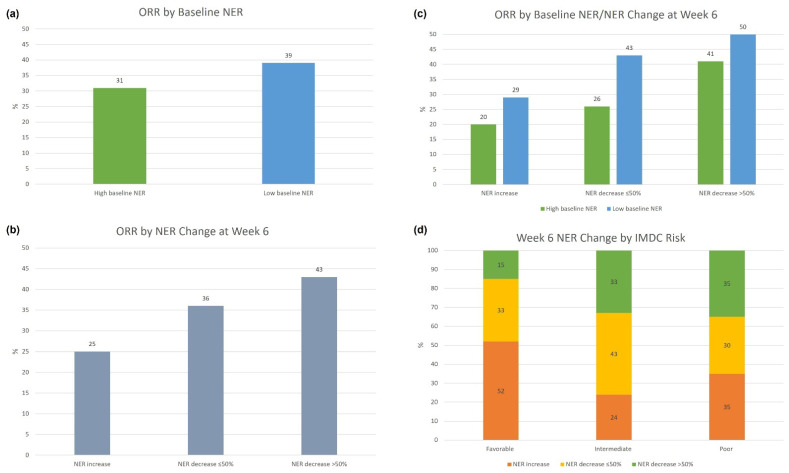
ORR by baseline NER and NER change at week 6.

For illustration purposes, baseline NER was subsequently dichotomized at the median (23.8) into high vs. low baseline NER groups, and the NER change from baseline at week 6 were grouped into three groups: (1) NER increase, (2) NER decrease ≤ 50%, and (3) NER decrease > 50%. When stratified by baseline NER (Figure 2a), patients with a low baseline NER had numerically higher ORR than those with a high baseline NER (39% vs. 31%, *p*-value: 0.30). When considering week 6 NER change alone (Figure 2b), NER decrease >50% had higher ORR compared to NER decrease ≤ 50% and NER increase (43% vs. 36% vs. 25%, *p*-value for trend: 0.07). When stratified by baseline NER and NER change at week 6 (Figure 2c), patients with NER decrease >50% at week 6 had numerically higher ORR compared to NER decrease ≤ 50% and NER increase in the low baseline NER subgroup (50% vs. 43% vs. 29%, *p*-value for trend: 0.16), and the high baseline NER subgroup (41% vs. 26% vs. 20%, *p*-value for trend: 0.11).When stratified by IMDC risk group (Figure 2d), patients with intermediate and poor risk disease had a higher percentage of NER decrease > 50% at week 6 compared to patients with favorable risk disease (33% and 35%, respectively vs. 15%, *p*-value: 0.048).

**Figure 3:**
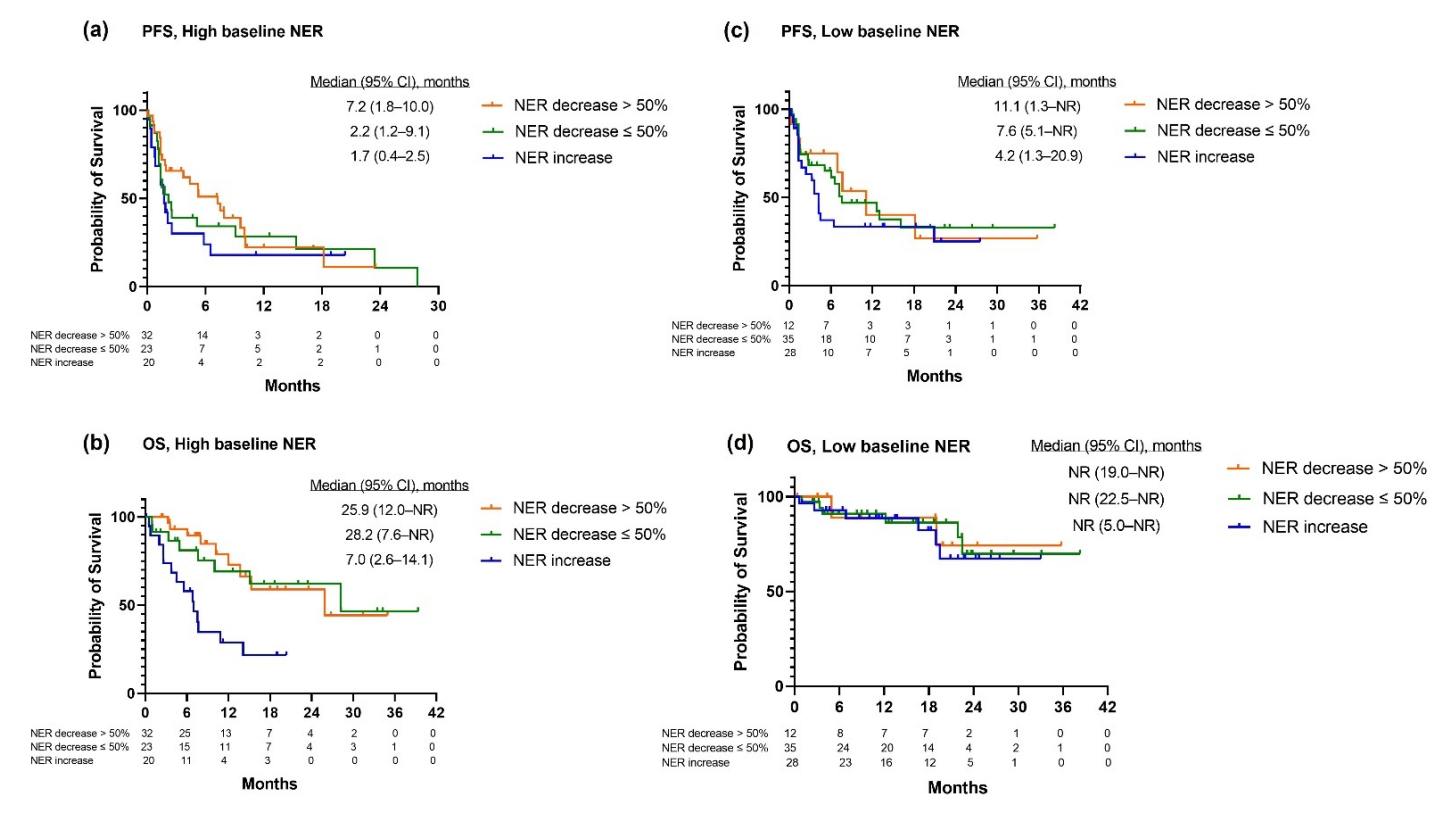
PFS/OS by baseline NER and NER change at week 6.

When NER was modeled by percent change, NER decrease > 50% at week 6 was associated with improved PFS (AHR:0.55, 95% CI: 0.31–0.95, *p*-value: 0.03) and OS (AHR: 0.37, 95% CI: 0.16–0.84, *p*-value: 0.02) when compared to increased NER (Table 2). NER decrease ≤50% at week 6 showed a trend toward improved PFS (AHR:0.63, 95% CI:0.38–1.05, *p*-value: 0.07) and OS (AHR: 0.49, 95% CI: 0.23–1.06, *p*-value: 0.07). Stratified analysis was conducted by baseline NER. In the subgroup with a high baseline NER, NER decrease > 50% was associated with improved PFS (AHR: 0.46, 95% CI: 0.22–1.00, *p*-value: 0.048) and OS (AHR: 0.28, 95% CI: 0.11–0.74, *p*-value: 0.01). In the subgroup with a low baseline NER, NER decrease > 50% at week 6 was not associated with PFS (AHR: 0.58, 95% CI: 0.22–1.48, *p*-value: 0.25) or OS (AHR: 0.60, 95% CI: 0.08–4.30, *p*-value: 0.61). The full models are provided in the Supplementary Material (Tables S1–S4). The Kaplan–Meier curves for PFS and OS by baseline NER and week 6 NER change are presented in Figure 3.

## 4. Discussion

The current study characterizes the dynamic changes of AEC and NER during the ipilimumab/nivolumab induction from baseline to week 12 in mRCC patients. Patients with an objective response had a higher interval increase in AEC, and there was a prominent decreasing trend of NER from baseline to week 12. After adjusting for patient sociodemographic and tumor characteristics, including the baseline NER [25] and IMDC prognostic risk [27], the decrease in NER at week 6 from the baseline was independently associated with improved PFS and OS in both the continuous and categorial variable analyses. In our subgroup analysis, this association was mostly driven by the subgroup with high baseline NER.

Building on our previous finding that baseline NER is predictive and prognostic in ipilimumab/nivolumab-treated mRCC [25], the current study demonstrates that the on-treatment decrease in NER at week 6 can be an early predictive biomarker of future response of ipilimumab/nivolumab, especially in patients with unfavorable high baseline NER. The current study observed a higher percentage of patients with NER decrease > 50% at week 6 in the IMDC intermediate risk (33%) and poor risk (35%) categories, compared to that in patients with favorable risk disease (15%). This finding is consistent with the CheckMate 214 [6], showing that the superior clinical efficacy of ipilimumab/nivolumab over sunitinib was mainly observed in the IMDC intermediate and poor risk, but not in the favorable risk categories. However, durable long-term responses have been observed among responders in the favorable risk group, but there is no available biomarker to select such patients. If the current biomarker is successfully validated in prospective studies, the decrease in on-treatment NER can be used as an early predictive biomarker in ipilimumab/nivolumab-treated mRCC.

These current findings are supported by previous studies that have observed the elevation of eosinophils after the initiation of ICIs, especially among patients with durable responses in lung cancer [21,22] and melanoma [23]. Prior to the ICI era, this positive association had been observed in IL-2-treated RCC in an early case series [30]. In addition to IO agents, the elevation of eosinophils was also observed in a post hoc analysis of three randomized-controlled phase III trials in sipuleucel-T-treated metastatic castration resistant prostate cancer (mCRPC) patients [31]: there was an association of an elevation of eosinophil at week 6 with improved prostate cancer-specific survival (HR: 0.71, 95% CI: 0.53–0.97, *p*-value: 0.03) and a trend for improved overall survival (HR: 0.75, 95% CI: 0.56–1.01, *p*-value: 0.06). The elevation of eosinophils after treatment initiation among responders suggests a certain biological effect of eosinophils in the TME. The anti-tumor activity of eosinophils has been postulated [32], and the roles of eosinophils in the TME have yet to be better elucidated [33]. Eosinophil can leave the bloodstream and migrate into the TME through several integrins-mediated mechanisms [34]. Pre-clinical studies [15,35,36] designed to mechanistically explicate the functions of eosinophils in the TME revealed enhanced antitumor responses through tumor-homing activated eosinophils: the activated eosinophils attracted tumor-specific CD8^+^ T cells by producing chemokines, such as CCL5, CXCL9, and CXCL10 [15]; the activated eosinophils also normalized the tumor vasculature and polarized the tumor-associated macrophages [15]; anti-tumor activities were also observed in eosinophil-mediated IL-33 [36] and the GM-CSF-IRF5 singling axis in melanoma [36] and colorectal cancer models [35], respectively.

Although our results showed an association between decreased NER and favorable outcomes among patients with ipilimumab/nivolumab-treated mRCC, there were still patients with increased NER at week 6 who had an objective response. Therefore, there are likely additional immune-mediated mechanisms at work in these patients. The current results reflect the complexity of the roles of innate immune cells in the TME, and further research is needed to untangle the involved biological pathways. Eosinophils have the plasticity to shape the TME in opposing directions, between pro-tumorigenic vs. anti-tumorigenic [33], depending on their reciprocal interactions with other cells [32]. Likewise, although tumor-associated neutrophils are traditionally considered to be associated with resistance to ICIs [37], studies revealed that the phenotypes of tumor-associated neutrophils were diverse, and their anti-tumor activities were observed in certain cancer types and at different disease stages [38,39,40]. Our study suggests the possibility of increasing the response of IO agents through enhancing the recruitment of eosinophils to the TME and the augmentation of the anti-tumor activities of the eosinophils. One of the potential targets may be the inhibition of the dipeptidyl peptidase 4 (DPP4) [41], which has been shown by Hollande et al. [42] to increase the chemokine CCL11-mediated eosinophil migration into breast cancer and hepatocellular carcinoma mouse models. In their study, DPP4 inhibition with sitagliptin was shown to increase eosinophil migration into the tumors, and the anti-tumor activity of eosinophils was shown to be independent of T-cells. Tumors treated with sitagliptin, in addition to anti-PD1/anti-CTLA4 antibodies, also demonstrated significantly lower tumor volume compared to controls. Our group previously conducted a small retrospective study of 26 patients with solid tumor diagnosis who were concomitantly taking DPP4 inhibitors for diabetes while on ICI treatment [43]: the objective response rate was 69% (18/26), although the results should be interpreted with caution due to the small sample size and lack of a comparison group. Of note, a phase 1b/2 trial investigating BXCL701, which is an inhibitor of DDP4/DPP8/DPP9, with/without pembrolizumab in mCRPC [44] (NCT03910660) reported a 26% response rate (6/23) and a disease control rate of 63% in this heavily treated patient population [45]. Future correlative studies of this trial may provide further translational data for the anti-tumor activity of eosinophils through DPP inhibitions and the possibility to enhance antitumor activity of ICIs.

In addition to improved clinical outcomes, the increase in eosinophil counts after initiation of ICIs was also reported to be an early biomarker for the development of immune-related adverse events (irAEs). Osawa et al. found that peripheral eosinophilia (defined as AEC ≥ 330/µL) at week 6 was associated with a 2.8-fold higher risk of irAEs [46]. Studies have observed such associations in ICI-induced adrenal insufficiency [47], hypopituitarism [48], cutaneous irAEs [49], and pneumonitis [50]. Given that irAEs have been associated with improved outcomes in ICI-treated cancer patients [51], the association between eosinophil elevation and irAEs is expected. The current dataset did not include granular information on irAEs; therefore, we did not explore such associations in our analysis.

There are several limitations in the current study. First, several peripheral blood count-based biomarkers have been previously investigated in various cancers, before and after the advent of ICIs, such as the neutrophil-to-lymphocyte ratio [52,53,54,55,56], lymphocyte-to-monocyte ratio [52,55,57], and platelet-to-lymphocyte ratio [52,54,58,59,60]. The current study chose to investigate NER (instead of other indices) based on our clinical observation of eosinophil count elevation and the established prognostic role of neutrophilia in mRCC in the IMDC model [27]. However, our results alone are insufficient to compare NER with other indices as a prognostic/predictive biomarker, as this study did not perform direct comparisons with each of these various indices. Prospective validation of NER is warranted. Second, the study design was a retrospective analysis, and inherited biases could not be avoided. The unmeasured confounding and residual confounding may still exist, despite the study-adopted regression method to adjust for baseline characteristics. Third, the median follow-up time was relatively short (11.6 months). Fourth, several other conditions, such as medications, infections, and the inflammatory/autoimmune process, can all affect neutrophil and eosinophil counts, and the current study was not able to account for those factors. As such, the results of the current study should be interpreted as hypothesis generating. Future clinical trials will provide a more ideal setting to prospectively validate the on-treatment NER biomarker. In addition, correlative studies in clinical trials can mechanistically explore the role of eosinophils in cancer immunotherapy by measuring baseline and on-treatment chemokines, such as CCL5/CXCL9/CXCL10/CCL11.

## 5. Conclusions

In patients with mRCC treated with ipilimumab/nivolumab, our results reveal that the decrease in on-treatment NER at week 6 is associated with improved PFS and overall survival, and the dynamic changes of on-treatment NER implied responses of ICIs. Prospective studies are warrantied to validate this predictive biomarker in IO combinations of mRCC and to further explore the role of eosinophils in TME, along with the therapeutic implications of eosinophil-mediated anti-tumor activities.

## Figures and Tables

**Table 1:** Patient baseline characteristics (N = 150).

	Median (IQR)
Baseline NER	23.8 (15–57)
Week 6 NER	19.8 (10.6–40.8)
	N (%)
Week 6 NER change	
Decrease > 50%	44 (29)
Decrease ≤ 50%	58 (39)
Increase	48 (32)
Baseline NER	
High (≥median)	75 (50)
Low (<median)	75 (50)
Age	(Median: 62 (IQR: 53–70))
<60	63 (42)
≥60	87 (58)
Sex	
Male	111 (74)
Female	39 (26)
Race	
White	120 (80)
Non-White	24 (14)
Unknown	9 (6)
Histology	
Clear cell	117 (78)
Non-clear cell	31 (21)
Unknown	2 (1)
IMDC risk	
Favorable	33 (22)
Intermediate	94 (63)
Poor	23 (15)
Nephrectomy	
Yes	104 (69)
No	46 (31)
Prior systemic therapy	
Yes	49 (33)
No	100 (67)
Unknown	1 (1)

NER: neutrophil-to-eosinophil ratio; IQR: interquartile range; IMDC: International Metastatic RCC Database Consortium.

**Table 2:** Multivariable regression analysis of NER and clinical outcomes.

	PFS		OS	
	AHR (95%)	*p*-Value	AHR (95%)	*p*-Value
Continuous variable				
Baseline LnNER	0.98 (0.78–1.23)	0.84	0.82 (0.57–1.19)	0.30
Week 6 LnNER	0.78 (0.66–0.93)	0.005	0.67 (0.52–0.86)	0.002
Week 6 NER change				
All patients (N = 150)				
Decrease > 50%	0.55 (0.31–0.95)	0.03	0.37 (0.16–0.84)	0.02
Decrease ≤ 50%	0.63 (0.38–1.05)	0.07	0.49 (0.23–1.06)	0.07
Increase	Ref		Ref	
Subgroup with high baseline NER (N = 75)				
Decrease > 50%	0.46 (0.22–1.00)	0.048	0.28 (0.11–0.74)	0.01
Decrease ≤ 50%	0.59 (0.26–1.31)	0.19	0.44 (0.16–1.23)	0.12
Increase	Ref		Ref	
Subgroup with low baseline NER (N = 75)				
Decrease > 50%	0.58 (0.22–1.48)	0.25	0.60 (0.08–4.30)	0.61
Decrease ≤ 50%	0.72 (0.34–1.53)	0.39	1.08 (0.25–4.70)	0.92
Increase	Ref		Ref	

PFS: progression-free survival; OS: overall survival; AHR: adjusted hazard ratio. Models were adjusted for age, sex, race, IMDC risk group, baseline NER, histology, prior systemic therapy, and prior nephrectomy. Landmark analysis for PFS and OS were calculated from week 6.

## Data Availability

The data presented in this study are available upon request. The data are not publicly available, as they contain information that could potentially compromise the privacy of the study subjects.

## Supplementary Materials

The following supporting information can be downloaded at: https://www.mdpi.com/article/10.3390/cancers14153830/s1, Table S1: continuous variable analysis for baseline LnNER and week 6 LnNER. Table S2: categorical variable analysis for week 6 NER change. Table S3: categorical variable analysis for week 6 NER change in subgroup with high baseline NER. Table S4: categorical variable analysis for week 6 NER change in subgroup with low baseline NER.
