# Supplementary material for: The Association between a Decrease in On-Treatment Neutrophil-to-Eosinophil Ratio (NER) at Week 6 after Ipilimumab Plus Nivolumab Initiation and Improved Clinical Outcomes in Metastatic Renal Cell Carcinoma
Source: Cancers (Basel). 2022 Aug 7;14(15):3830. doi: 10.3390/cancers14153830 (PMC9367298; doi:10.3390/cancers14153830)
Supplement: Supplementary file 1 [file cancers-14-03830-s001.zip › cancers-1803593-supplementary.pdf]

Supplementary table S1: continuous variable analysis for baseline LnNER and week 6 LnNER

	PFS		OS	
	**AHR (95%)**	**P-value**	**AHR (95%)**	**P-value**
**Baseline LnNER**	0.98 (0.78-1.23)	0.84	0.82 (0.57-1.19)	0.30
**Week 6 LnNER**	0.78 (0.66-0.93)	0.005	0.67 (0.52-0.86)	0.002
**Histology**				
ccRCC	1.12 (0.64-1.98)	0.69	0.52 (0.24-1.11)	0.09
nccRCC	Ref		Ref	
**Prior Systemic Therapy**				
Yes	1.86 (1.19-2.91)	0.006	1.37 (0.67-2.82)	0.39
No	Ref		Ref	
**Age**				
≥60	0.89 (0.58-1.38)	0.61	0.97 (0.48-1.95)	0.93
<60	Ref		Ref	
**Sex**				
Male	1.18 (0.74-1.89)	0.48	1.42 (0.68-2.96)	0.35
Female	Ref		Ref	
**Race**				
White	0.67 (0.36-1.24)	0.20	0.55 (0.22-1.36)	0.20
Non-White	Ref		Ref	
**Nephrectomy**				
Yes	0.64 (0.39-1.07)	0.09	0.52 (0.24-1.12)	0.09
No	Ref		Ref	
**IMDC risk**				
Poor	2.27 (1.06-4.87)	0.03	5.60 (1.69-18.6)	0.005
Intermediate	1.85 (1.07-3.20)	0.03	2.21 (0.80-6.05)	0.12
Favorable	Ref		Ref	

Supplementary table S2: categorical variable analysis for week 6 NER change

	PFS		OS	
	**AHR (95%)**	**P-value**	**AHR (95%)**	**P-value**
**Week 6 NER change**				
Decrease >50%	0.55 (0.31-0.95)	0.03	0.37 (0.16-0.84)	0.02
Decrease ≤50%	0.63 (0.38-1.05)	0.07	0.49 (0.23-1.06)	0.07
Increase	Ref		Ref	
**Histology**				
ccRCC	1.11 (0.61-2.00)	0.74	0.54 (0.24-1.20)	0.13
nccRCC	Ref		Ref	
**Prior Systemic Therapy**				
Yes	1.78 (1.14-2.76)	0.01	1.22 (0.59-2.52)	0.59
No	Ref		Ref	
**Age**				
≥60	0.86 (0.55-1.33)	0.49	0.93 (0.47-1.86)	0.84
<60	Ref		Ref	
**Sex**				
Male	1.14 (0.72-1.81)	0.57	1.35 (0.65-2.78)	0.42
Female	Ref		Ref	
**Race**				
White	0.68 (0.36-1.26)	0.22	0.60 (0.24-1.50)	0.28
Non-White	Ref		Ref	
**Nephrectomy**				
Yes	0.67 (0.40-1.13)	0.13	0.53 (0.24-1.15)	0.11
No	Ref		Ref	
**IMDC risk**				
Poor	2.07 (0.97-4.40)	0.06	5.44 (1.65-18.0)	0.005
Intermediate	1.71 (0.99-2.95)	0.05	2.23 (0.80-6.22)	0.13
Favorable	Ref		Ref	

Supplementary table S3: categorical variable analysis for week 6 NER change in subgroup with high baseline NER

	PFS		OS	
	**AHR (95%)**	**P-value**	**AHR (95%)**	**P-value**
**Week 6 NER change**				
Decrease >50%	0.46 (0.22-1.00)	0.048	0.28 (0.11-0.74)	0.01
Decrease ≤50%	0.59 (0.26-1.31)	0.19	0.44 (0.16-1.23)	0.12
Increase	Ref		Ref	
**Histology**				
ccRCC	1.38 (0.57-3.37)	0.48	0.62 (0.22-1.75)	0.37
nccRCC	Ref		Ref	
**Prior Systemic Therapy**				
Yes	2.60 (1.38-4.91)	0.003	1.05 (0.41-2.72)	0.92
No	Ref		Ref	
**Age**				
≥60	0.36 (0.19-0.70)	0.003	0.53 (0.21-1.32)	0.17
<60	Ref		Ref	
**Sex**				
Male	1.10 (0.57-2.11)	0.78	0.88 (0.38-2.06)	0.77
Female	Ref		Ref	
**Race**				
White	0.71 (0.28-1.81)	0.47	0.55 (0.17-1.76)	0.31
Non-White	Ref		Ref	
**Nephrectomy**				
Yes	0.62 (0.31-1.22)	0.17	0.64 (0.26-1.60)	0.34
No	Ref		Ref	
**IMDC risk**				
Poor	5.07 (1.38-18.6)	0.01	NE	
Intermediate	2.55 (0.88-7.36)	0.08	NE	
Favorable	Ref		Ref	

NE: not-estimable due to small number of events

Supplementary table S4: categorical variable analysis for week 6 NER change in subgroup with low baseline NER

	PFS		OS	
	**AHR (95%)**	**P-value**	**AHR (95%)**	**P-value**
**Week 6 NER change**				
Decrease >50%	0.58 (0.22-1.48)	0.25	0.60 (0.08-4.30)	0.61
Decrease ≤50%	0.72 (0.34-1.53)	0.39	1.08 (0.25-4.70)	0.92
Increase	Ref		Ref	
**Histology**				
ccRCC	0.95 (0.31-2.96)	0.94	0.20 (0.04-1.10)	0.06
nccRCC	Ref		Ref	
**Prior Systemic Therapy**				
Yes	2.03 (0.94-4.38)	0.07	1.76 (0.40-7.69)	0.45
No	Ref		Ref	
**Age**				
≥60	1.85 (0.91-3.76)	0.09	2.23 (0.52-9.62)	0.28
<60	Ref		Ref	
**Sex**				
Male	1.09 (0.47-2.54)	0.84	1.69 (0.18-16.1)	0.65
Female	Ref		Ref	
**Race**				
White	0.47 (0.17-1.31)	0.15	0.48 (0.05-4.54)	0.52
Non-White	Ref		Ref	
**Nephrectomy**				
Yes	0.59 (0.23-1.48)	0.26	0.34 (0.07-1.75)	0.20
No	Ref		Ref	
**IMDC risk**				
Poor	1.42 (0.41-4.95)	0.58	1.92 (0.16-23.6)	0.61
Intermediate	1.82 (0.89-3.73)	0.10	1.60 (0.42-6.08)	0.49
Favorable	Ref		Ref	
